# The effect of dietary supplementation with silkworm pupae meal on gastrointestinal function, nitrogen retention and blood biochemical parameters in rabbits

**DOI:** 10.1186/s12917-021-02906-w

**Published:** 2021-06-02

**Authors:** Andrzej Gugołek, Dorota Kowalska, Janusz Strychalski, Katarzyna Ognik, Jerzy Juśkiewicz

**Affiliations:** 1 Department of Fur-bearing Animal Breeding and Game Management, Faculty of Animal Bioengineering, University of Warmia and Mazury in Olsztyn, Oczapowskiego 5, 10-718 Olsztyn, Poland; 2 Department of Small Livestock Breeding, National Research Institute of Animal Production, 32-083 Balice n. Kraków, Poland; 3 Department of Biochemistry and Toxicology, Faculty of Biology, Animal Sciences and Bioeconomy, University of Life Sciences in Lublin, Akademicka 13, 20-950 Lublin, Poland; 4 Institute of Animal Reproduction and Food Research, Polish Academy of Sciences, Tuwima 10, 10-748 Olsztyn, Poland

**Keywords:** Rabbits, Silkworm pupae meal, Nutrient digestibility, Nitrogen retention, Gastrointestinal function, Blood biochemical parameters

## Abstract

**Background:**

The aim of this study was to determine the effect of dietary inclusion of silkworm pupae meal (SPM) on nutrient digestibility, nitrogen utilization, gastrointestinal physiology and blood biochemical parameters in rabbits. Thirty Termond White rabbits were divided into three groups: SBM – fed a diet containing 10% soybean meal (SBM), SPM5 – fed a diet containing 5% SBM and 5% SPM, and SPM10 – fed a diet containing 10% SPM.

**Results:**

Nutrient digestibility and nitrogen retention decreased with increasing SPM inclusion levels in rabbit diets. The dietary inclusion of SPM caused a significant increase in the stomach pH. Group SPM10 rabbits were characterized by the highest cecal tissue and digesta weights. The lowest cecal pH was noted in group SPM5. The relative weights of colonic tissue and digesta tended to increase with increasing levels of SPM. The total and intracellular activity of bacterial α-galactosidase decreased significantly in both SPM groups. The replacement of SBM with SPM led to a decrease in the activity of bacterial β-glucuronidase in the cecal digesta. The intracellular activity of bacterial α-arabinofuranosidase increased, and its release rate decreased in the cecum of rabbits in SPM groups. The extracellular activity of bacterial β-xylosidase in the cecal digesta tended to decrease in group SPM10. The highest extracellular and intracellular activity of bacterial β-cellobiosidase in the cecal digesta was noted in the SPM5 treatment. The lowest and the highest activity of bacterial N-acetyl-β-D-glucosaminidase (NAGase) was observed in groups SBM and SPM10. The SPM10 treatment contributed to a decrease in the cecal concentrations of butyric, iso-valeric and valeric acids. The lowest total concentration of putrefactive short-chain fatty acids (PSCFAs) was observed in group SPM10. The cecal concentration of propionic acid tended to increase in group SPM5, whereas the cecal concentration of iso-butyric acid tended to decrease in group SPM10. The colonic concentration of iso-valeric acid was lowest in group SPM5. SPM treatments resulted in a significant increase in plasma albumin concentration. Plasma urea concentration was significantly higher in group SPM10 than in SBM and SPM5.

**Conclusions:**

The results of this study suggest that rabbit diets can be supplemented with SPM at up to 5%.

## Background

European rabbits (*Oryctolagus cuniculus*) are non-ruminant herbivores. They have a simple, non-compartmentalized stomach along with an enlarged cecum and colon inhabited by a microbial population, primarily Bacteroides. Microbes digest cellulose mostly in the hindgut of rabbits. Moreover, rabbits practice coprophagy, which also increases protein digestibility. Feces are excreted on a circadian rhythm, and published data indicate that the internal cycle differs when shifting from ad libitum to restricted feeding. The gastrointestinal system of rabbits is unique, and adequate nutrition and intestinal microbial balance are required to maintain normal gastrointestinal tract (GIT) function. An important role is played by the large intestinal environment, which is the main site of bacterial fermentation [1, 2].

Complete balanced diets for growing rabbits should contain considerable amounts of fiber and protein [3]. At present, soybean meal (SBM) is the main source of protein in diets for livestock, including rabbits. Research has shown that in rabbit diets, SBM can be replaced with food processing by-products such as rapeseed cake, dried distillers grains and legume seeds [4–8].

Attempts have also been made to supplement rabbit diets with animal protein sources, including dairy products, fishmeal, meat-and-bone meal and poultry by-product meal [9–13]. However, it should be noted that the use of most meat-and-bone meals in animal feeds has been banned in many countries (refer e.g. to Regulation (*EC*) No *999/2001* of the *European Parliament* and of the Council of 22 *May 2001*).

In recent years, insect meals have been considered as the most promising alternative sources of animal protein. Their efficacy has been investigated in various livestock species, mostly poultry. The group of insect meals includes silkworm (*Bombyx mori*) pupae meal (SPM), the by-product of sericulture, which contains more than 20% of fat and more than 50% of protein with relatively high concentrations of nutritionally valuable amino acids such as lysine and methionine [14–16].

In a study by Liu et al. [17], silkworm pupae were not analyzed as an experimental factor but as a component of rabbit diets, which points to their common use in China. The use of SPM as a substitute for SBM in rabbit diets was described by Carregal and Takahashi [18], Gugołek et al. [19] and Kowalska et al. [20]. The cited authors focused on the effects of SPM on animal performance and the chemical composition of meat. The results of the above studies indicate that SPM can be included in rabbit diets at up to 5% without compromising productivity. The influence of SPM on GIT function and the health status of rabbits has not been evaluated to date. Insect meals are not natural components of rabbit diets, which suggests that they may have negative health consequences such as subclinical states which are difficult to identify based on performance parameters. It should be stressed that insects contain chitin, which is indigestible by rabbits [21], as well as other unidentified factors whose effects on rabbits are unknown.

It is well known that the mammalian gut microbiome plays a very important role in metabolic, nutritional, physiological and immunological processes [22], and the productivity of farm animals [23, 24]. In rabbits, most gastrointestinal disorders are associated with inadequate nutrition and manifest as dysbiosis, i.e. disruption of intestinal microbiota homeostasis. Dysbiosis predisposes rabbits to the enteritis complex, a major cause of morbidity and mortality [2, 25].

Since the influence of insect protein sources on growing rabbits has not been thoroughly investigated, the aim of this study was to determine the effect of different dietary levels of dried SPM on gastrointestinal function in rabbits, with particular emphasis on nutrient digestibility and enzyme activity in different GIT segments. Nitrogen retention and selected blood biochemical parameters were also analyzed.

## Results

The inclusion of SPM in rabbit diets had no significant effect on the digestibility coefficients of DM, organic matter, crude protein, NDF, ADF, ADL and energy (Table 1). However, the values of digestibility coefficients tended to decrease with increasing dietary inclusion levels of SPM. Such a relationship was not observed for ether extract whose digestibility coefficients were highly similar in the control group (SBM) and both experimental groups (SPM5 and SPM10).

**Table 1:** Nutrient digestibility and nitrogen (N) utilization in rabbits (mean ± SEM^1^)

	Group^2^			*p value*
	SBM	SPM5	SPM10	
Digestibility [%]				
Dry matter	62.61 ± 0.99	61.03 ± 3.21	58.31 ± 1.75	*0.260*
Organic matter	64.04 ± 1.00	62.56 ± 5.28	59.65 ± 3.29	*0.279*
Crude protein	73.75 ± 0.92	73.18 ± 2.17	70.61 ± 1.07	*0.232*
Ether extract	80.68 ± 1.44	80.77 ± 5.87	80.98 ± 1.65	*0.955*
Neutral detergent fiber	40.34 ± 1.66	38.99 ± 5.26	33.78 ± 3.45	*0.159*
Acid detergent fiber	30.05 ± 2.04	29.34 ± 6.31	25.51 ± 8.32	*0.246*
Acid detergent lignin	34.13 ± 3.28	31.30 ± 4.41	28.49 ± 5.76	*0.253*
Gross energy	63.52 ± 1.81	61.80 ± 3.17	59.62 ± 1.72	*0.196*
Daily N balance [g/rabbit]				
Intake	3.98 ± 0.08^a^	3.60 ± 0.10^ab^	2.99 ± 0.23^b^	*0.005*
Excretion with feces	1.15 ± 0.05	1.08 ± 0.07	0.90 ± 0.12	*0.164*
Excretion with urine	0.72 ± 0.05^a^	0.68 ± 0.10^ab^	0.57 ± 0.11^b^	*0.018*
Digestion	2.83 ± 0.04^a^	2.52 ± 0.10^ab^	2.09 ± 0.14^b^	*0.003*
Retention	2.11 ± 0.11^a^	1.84 ± 0.10^ab^	1.52 ± 0.11^b^	*0.042*
N retention [%]				
Relative to N intake	53.01 ± 4.64	51.11 ± 4.57	50.84 ± 5.91	*0.724*
Relative to N digested	74.56 ± 6.27	73.01 ± 6.04	72.73 ± 6.24	*0.862*

^a, b^means with different superscripts in the same row differ significantly (*P* < 0.05)

^1^SEM - standard error of the mean

^2^Group: SBM - 10% SBM, SPM5–5% SBM and 5% SPM, SPM10–10% SPM (SBM - soybean meal, SPM - dried silkworm pupae meal)

Nitrogen intake, excretion in feces, excretion in urine, digestion and retention decreased with increasing inclusion levels of SPM. However, significant (*P* < 0.05) differences were noted only for N intake, urinary excretion, digestion and retention between the control group (SBM) and the second experimental group (SPM10). Nitrogen retention relative to N intake and N digested did not differ significantly between groups, but the calculated values were highest in group SBM and lowest in group SPM10.

The dietary inclusion of SPM at both levels caused a significant increase in the pH of stomach digesta (*P* < 0.05 vs. SBM, Table 2). The applied dietary treatments did not affect the relative weights of small intestinal tissue and digesta, digesta viscosity rate or pH (*P* > 0.05). The percentage of jejunal digesta hydration tended to increase in SPM treatments (*P* = 0.063). Group SPM10 rabbits were characterized by the highest relative weights of cecal tissue and digesta (*P* < 0.05 vs. SBM and *P* < 0.05 vs. SBM, SPM5, respectively). The lowest cecal pH value was noted in group SPM5 (*P* < 0.05 vs. SPM10). The relative weights of colonic tissue and digesta tended to increase with increasing dietary inclusion levels of SPM (*P* = 0.090 and *P* = 0.068, respectively).

**Table 2:** Selected gastrointestinal tract parameters in rabbits (mean ± SEM^1^)

	Group^2^			*p value*
	SBM	SPM5	SPM10	
**Stomach**				
pH of digesta	1.98 ± 0.093^b^	2.50 ± 0.093^a^	2.54 ± 0.116^a^	*0.002*
**Small intestine**				
Tissue weight [g/kg BW]	22.8 ± 1.611	24.6 ± 0.926	25.4 ± 1.004	*0.193*
Digesta weight [g/kg BW]	14.6 ± 0.882	14.1 ± 0.626	17.2 ± 1.491	*0.083*
Viscosity [mPa·s]	5.89 ± 0.413	5.45 ± 0.434	5.23 ± 0.293	*0.296*
DM of jejunal digesta [%]	14.6 ± 1.417	12.8 ± 0.770	11.6 ± 0.470	*0.063*
pH of jejunal digesta	7.16 ± 0.063	7.27 ± 0.041	7.11 ± 0.083	*0.155*
**Cecum**				
Tissue weight [g/kg BW]	10.3 ± 0.338^b^	11.4 ± 0.300^ab^	11.9 ± 0.462^a^	*0.012*
Digesta weight [g/kg BW]	38.7 ± 1.956^b^	43.8 ± 2.836^b^	55.8 ± 3.319^a^	*< 0.001*
DM of digesta [%]	22.9 ± 0.685	23.7 ± 0.282	23.4 ± 0.624	*0.377*
Ammonia [mg/g]	0.351 ± 0.011	0.342 ± 0.015	0.347 ± 0.013	*0.684*
pH of digesta	6.67 ± 0.046^ab^	6.56 ± 0.072^b^	6.79 ± 0.041^a^	*0.013*
**Colon**				
Tissue weight [g/kg BW]	9.89 ± 0.366	10.5 ± 0.403	11.2 ± 0.574	*0.090*
Digesta weight [g/kg BW]	10.8 ± 0.688	12.7 ± 1.216	14.6 ± 1.627	*0.068*
pH of digesta	6.57 ± 0.080	6.61 ± 0.094	6.57 ± 0.146	*0.829*

^a, b^means with different superscripts in the same row differ significantly (*P* < 0.05)

^1^SEM - standard error of the mean

^2^Group: SBM - 10% SBM, SPM5–5% SBM and 5% SPM, SPM10–10% SPM (SBM - soybean meal, SPM - dried silkworm pupae meal)

The extracellular activity of bacterial α-glucosidase in the cecal digesta was significantly lower whereas the intracellular activity of this enzyme was significantly higher in group SPM10 than in groups SBM and SPM5 (Table 3). The release rate of cecal α-glucosidase was lowest in group SPM10 (*P* < 0.05 vs. the other treatments). The extracellular and total activity of bacterial β-glucosidase in the cecal digesta was lowest in the SPM10 treatment (*P* < 0.05 vs. SBM and SPM5). The intracellular activity of β-glucosidase in the cecal digesta increased in group SPM5 (*P* < 0.05 vs. SBM). In comparison with group SBM, the release rate of bacterial β-glucosidase in the cecum was significantly reduced in both groups fed SPM diets, but the lowest release percentage was noted in the SPM10 treatment (*P* < 0.05 vs. SBM and SPM5). The lowest and highest extracellular activity of cecal α-galactosidase was observed in treatments SPM10 and SBM, respectively (in both cases *P* < 0.05 vs. the other groups). Both groups fed SPM diets had significantly reduced total and intracellular activity of bacterial α-galactosidase, compared with the control group (SBM). Complete replacement of SBM with SPM in diets caused a significant decrease in the total and extracellular activity of bacterial β-glucuronidase in the cecal digesta vs. the control group (SBM).

**Table 3:** Activity of bacterial α- and β-glucosidase, α- and β-galactosidase, and β-glucuronidase [μmol/h/g digesta] in the cecal digesta of rabbits (mean ± SEM^1^)

	Group^2^			*p value*
	SBM	SPM5	SPM10	
α-Glucosidase				
Extracellular	6.14 ± 0.626^a^	5.14 ± 0.545^a^	3.06 ± 0.202^b^	*< 0.001*
Intracellular	0.520 ± 0.152^b^	0.650 ± 0.080^b^	2.21 ± 0.304^a^	*< 0.001*
Total	6.66 ± 0.757	5.79 ± 0.584	5.27 ± 0.215	*0.137*
Release rate^3^	93.0 ± 1.149^a^	88.4 ± 1.481^a^	58.9 ± 4.563^b^	*< 0.001*
β-Glucosidase				
Extracellular	5.79 ± 0.626^a^	4.26 ± 0.591^a^	1.77 ± 0.217^b^	*< 0.001*
Intracellular	2.26 ± 0.362^b^	3.64 ± 0.507^a^	2.99 ± 0.234^ab^	*0.033*
Total	8.05 ± 0.724^a^	7.90 ± 0.984^a^	4.76 ± 0.301^b^	*0.010*
Release rate^3^	71.9 ± 3.565^a^	53.5 ± 3.271^b^	36.9 ± 3.854^c^	*< 0.001*
α-Galactosidase				
Extracellular	8.21 ± 0.631^a^	5.77 ± 0.398^b^	3.09 ± 0.328^c^	*< 0.001*
Intracellular	7.37 ± 0.583^a^	4.36 ± 0.213^b^	5.17 ± 0.635^b^	*0001*
Total	15.6 ± 1.051^a^	10.1 ± 0.554^b^	8.26 ± 0.668^b^	*< 0.001*
Release rate^3^	52.7 ± 2.104^a^	56.6 ± 1.589^a^	39.2 ± 5.022^b^	*0.002*
β-Galactosidase				
Extracellular	11.8 ± 1.665	11.2 ± 1.303	9.13 ± 0.858	*0.221*
Intracellular	14.7 ± 1.998	12.5 ± 2.972	12.1 ± 1.777	*0.476*
Total	26.5 ± 3.526	23.7 ± 3.832	21.2 ± 2.157	*0.317*
Release rate^3^	44.4 ± 2.115	49.6 ± 3.620	44.5 ± 3.240	*0.287*
β-Glucuronidase				
Extracellular	36.7 ± 4.006^a^	32.5 ± 3.150^ab^	23.5 ± 2.000^b^	*0.015*
Intracellular	32.8 ± 2.675	28.2 ± 5.291	24.7 ± 2.511	*0.189*
Total	69.5 ± 4.877^a^	60.7 ± 5.947^ab^	48.2 ± 3.262^b^	*0.011*
Release rate^3^	52.2 ± 3.548	55.2 ± 4.127	49.1 ± 3.449	*0.319*

^a, b, c^means with different superscripts in the same row differ significantly (*P* < 0.05)

^1^SEM - standard error of the mean

^2^Group: SBM - 10% SBM, SPM5–5% SBM and 5% SPM, SPM10–10% SPM (SBM - soybean meal, SPM - dried silkworm pupae meal)

^3^Release rate = extracellular enzyme activity expressed as a percentage of total (extra- + intracellular) enzyme activity

The extracellular activity of bacterial α-arabinopyranosidase was significantly reduced, whereas its intracellular activity was enhanced in the SPM5 treatment (*P* < 0.05 vs. SBM and *P* < 0.05 vs. SBM, respectively, Table 4). The release rate of α-arabinopyranosidase decreased in response to the total replacement of SBM with SPM (*P* < 0.05 vs. SBM and SPM5). The SPM5 treatment contributed to a decrease in the extracellular activity of cecal α-arabinofuranosidase (*P* < 0.05 vs. the other groups). In both groups fed SPM diets, the intracellular activity of bacterial α-arabinofuranosidase increased significantly and its release rate decreased in the cecum, compared with the control group (SBM). A tendency towards lower extracellular activity of bacterial β-xylosidase in the cecal digesta was observed in rabbits fed the SPM10 diet (*P* = 0.079). The intracellular activity of bacterial β-xylosidase was highest in group SPM5 (*P* < 0.05 vs. SBM). The release rate of this enzyme from bacterial cells into the cecal environment was significantly lower in both SPM groups than in the control group (SBM). The highest bacterial extracellular and intracellular activity of β-cellobiosidase in the cecal digesta was noted in the SPM5 treatment (*P* < 0.05 vs. SPM10 and *P* < 0.05 vs. SBM, respectively). The release rate of cecal β-cellobiosidase was lower in treatments SPM5 and SPM10 than in the control group (SBM). The lowest and highest extracellular activity of bacterial N-acetyl-β-D-glucosaminidase (NAGase) was observed in groups SBM and SPM10, respectively (in both cases *P* < 0.05 vs. the other treatments). Similar differences among groups were noted for the release rate of NAGase in the cecum. In addition, group SPM10 rabbits were characterized by the highest total activity of bacterial NAGase in the cecum (*P* < 0.05 vs. SBM).

**Table 4:** Activity of bacterial α-arabinopyranosidase, α-arabinofuranosidase, β-xylosidase, β-cellobiosidase and N-acetyl-β-D-glucosaminidase [μmol/h/g digesta] in the cecal digesta of rabbits (mean ± SEM^1^)

	Group^2^			*p value*
	SBM	SPM5	SPM10	
α-Arabinopyranosidase				
Extracellular	2.06 ± 0.179^ab^	2.43 ± 0.444^a^	1.21 ± 0.241^b^	*0.021*
Intracellular	0.413 ± 0.166^b^	0.692 ± 0.147^ab^	1.06 ± 0.191^a^	*0.025*
Total	2.47 ± 0.262	3.12 ± 0.577	2.27 ± 0.397	*0.229*
Release rate^3^	85.6 ± 4.508^a^	79.0 ± 2.666^a^	53.6 ± 4.730^b^	*< 0.001*
α-Arabinofuranosidase				
Extracellular	2.40 ± 0.306^a^	2.55 ± 0.466^a^	1.27 ± 0.253^b^	*0.032*
Intracellular	0.281 ± 0.117^b^	1.20 ± 0.172^a^	1.11 ± 0.247^a^	*0.005*
Total	2.68 ± 0.345	3.75 ± 0.521	2.37 ± 0.444	*0.064*
Release rate^3^	90.4 ± 3.474^a^	65.5 ± 4.946^b^	55.6 ± 6.836^b^	*< 0.001*
β-Xylosidase				
Extracellular	2.83 ± 0.629	2.97 ± 0.542	1.48 ± 0.294	*0.079*
Intracellular	0.541 ± 0.161^b^	1.75 ± 0.394^a^	1.52 ± 0.348^ab^	*0.025*
Total	3.38 ± 0.780	4.72 ± 0.736	3.00 ± 0.513	*0.131*
Release rate^3^	83.0 ± 2.713^a^	64.5 ± 5.663^b^	52.4 ± 7.775^b^	*0.002*
β-Cellobiosidase				
Extracellular	1.90 ± 0.237^ab^	2.09 ± 0.350^a^	1.07 ± 0.185^b^	*0.025*
Intracellular	0.476 ± 0.142^b^	1.54 ± 0.346^a^	1.34 ± 0.306^ab^	*0.024*
Total	2.03 ± 0.238	2.72 ± 0.378	1.72 ± 0.332	*0.062*
Release rate^3^	93.3 ± 1.390^a^	74.2 ± 3.721^b^	65.2 ± 4.862^b^	*< 0.001*
NAGase				
Extracellular	3.13 ± 0.560^c^	5.30 ± 0.399^b^	7.04 ± 0.513^a^	*< 0.001*
Intracellular	4.36 ± 0.474	4.78 ± 0.594	4.02 ± 0.498	*0.378*
Total	7.48 ± 0.969^b^	10.1 ± 0.902^ab^	11.1 ± 0.691^a^	*0.016*
Release rate^3^	40.2 ± 2.541^c^	53.2 ± 2.211^b^	64.1 ± 3.174^a^	*< 0.001*

^a, b, c^means with different superscripts in the same row differ significantly (*P* < 0.05)

^1^SEM - standard error of the mean

^2^Group: SBM - 10% SBM, SPM5–5% SBM and 5% SPM, SPM10–10% SPM (SBM - soybean meal, SPM - dried silkworm pupae meal)

^3^Release rate = extracellular enzyme activity expressed as a percentage of total (extra- + intracellular) enzyme activity

The extracellular and total activity of bacterial α-glucosidase in the colonic digesta was significantly reduced in the SPM10 treatment (*P* < 0.05 vs. SBM and *P* < 0.05 vs. SBM, SPM5, respectively, Table 5). Both SPM treatments caused a decrease in the extracellular activity of α-galactosidase and β-glucuronidase in the colonic digesta, compared with group SBM (*P* < 0.05). In addition, the total activity of bacterial α-galactosidase was significantly lower in groups SPM5 and SPM10, and a strong statistical tendency towards reduced total activity of bacterial β-glucuronidase was noted in the above treatments relative to the control group (SBM). The release rate of bacterial α-galactosidase into the colonic environment tended to decrease in dietary treatments SPM5 and SPM10 vs. SBM (*P* = 0.051). Complete replacement of SBM with SPM led to a significant increase in the extracellular activity of colonic bacterial β-cellobiosidase and the total activity of NAGase in comparison with group SBM (*P* < 0.05). Both SPM treatments (groups SPM5 and SPM10) significantly increased the extracellular activity of bacterial NAGase and the release rates of bacterial β-cellobiosidase and NAGase in the colonic digesta relative to the control group (SBM).

**Table 5:** Bacterial enzyme activity[μmol/h/g digesta] in the colonic digesta of rabbits (mean ± SEM^1^)

	Group^2^			*p value*
	SBM	SPM5	SPM10	
α-Glucosidase				
Extracellular	2.31 ± 0.273^a^	2.07 ± 0.182^ab^	1.50 ± 0.123^b^	*0.018*
Intracellular	1.38 ± 0.259	1.40 ± 0.192	1.03 ± 0.100	*0.241*
Total	3.70 ± 0.225^a^	3.47 ± 0.292^a^	2.53 ± 0.134^b^	*0.003*
Release rate^3^	62.6 ± 6.190	60.3 ± 3.227	59.3 ± 3.604	*0.658*
α-Galactosidase				
Extracellular	3.89 ± 0.385^a^	2.56 ± 0.276^b^	1.73 ± 0.169^b^	*< 0.001*
Intracellular	4.62 ± 0.478	4.16 ± 0.366	3.53 ± 0.466	*0.135*
Total	8.51 ± 0.419^a^	6.70 ± 0.613^b^	5.26 ± 0.432^b^	*< 0.001*
Release rate^3^	46.0 ± 4.475	37.9 ± 1.381	34.5 ± 3.874	*0.051*
β-Glucuronidase				
Extracellular	13.4 ± 1.263^a^	9.83 ± 1.159^b^	9.32 ± 0.797^b^	*0.028*
Intracellular	38.4 ± 2.579	33.4 ± 2.343	36.2 ± 3.123	*0.263*
Total	51.8 ± 2.972	43.2 ± 1.468	45.5 ± 3.131	*0.054*
Release rate^3^	26.0 ± 2.106	23.4 ± 3.512	21.3 ± 2.294	*0.293*
β-Cellobiosidase				
Extracellular	0.235 ± 0.037^b^	0.383 ± 0.065^ab^	0.491 ± 0.074^a^	*0.014*
Intracellular	0.357 ± 0.052	0.281 ± 0.041	0.349 ± 0.055	*0.344*
Total	0.592 ± 0.056	0.663 ± 0.098	0.840 ± 0.120	*0.117*
Release rate^3^	40.7 ± 5.621^b^	55.6 ± 4.220^a^	57.4 ± 4.332^a^	*0.038*
NAGase				
Extracellular	1.01 ± 0.073^b^	1.94 ± 0.253^a^	2.51 ± 0.302^a^	*< 0.001*
Intracellular	1.98 ± 0.332	1.99 ± 0.162	2.05 ± 0.359	*0.897*
Total	2.99 ± 0.372^b^	3.92 ± 0.391^ab^	4.55 ± 0.468^a^	*0.027*
Release rate^3^	36.7 ± 3.457^b^	48.6 ± 2.239^a^	55.9 ± 4.314^a^	*0.002*

^a, b^means with different superscripts in the same row differ significantly (*P* < 0.05)

^1^SEM - standard error of the mean

^2^Group: SBM - 10% SBM, SPM5–5% SBM and 5% SPM, SPM10–10% SPM (SBM - soybean meal, SPM - dried silkworm pupae meal)

^3^Release rate = extracellular enzyme activity expressed as a percentage of total (extra- + intracellular) enzyme activity

The SPM10 treatment caused a significant decrease in the cecal concentrations of butyric, iso-valeric and valeric acids (*P* < 0.05 vs. SPM5, *P* < 0.05 vs. SBM, SPM5, *P* < 0.05 vs. SPM5, respectively). The lowest total concentration of PSCFAs was observed in group SPM10 (*P* < 0.05 vs. SBM, SPM5, Table 6). A statistical tendency towards increased cecal concentration of propionic acid was noted in group SPM5, whereas the cecal concentration of iso-butyric acid tended to decrease in the SPM10 treatment (*P* = 0.055 and *P* = 0.067, respectively). The total concentration of SCFAs in the cecal digesta was not affected by the applied dietary treatments, but the cecal SCFA pool, i.e. the sum of SCFA concentrations and the bulk of digesta in the cecum, tended to decrease in rabbits fed SPM diets (*P* = 0.067). An analysis of the SCFA profile revealed the highest proportion of acetic acid and the lowest proportion of butyric acid in group SPM10 (in both cases *P* < 0.05 vs. SPM5). In the colonic digesta, the total concentrations of SCFAs and acetic acid tended to decrease in the SPM10 treatment (*P* = 0.053 and *P* = 0.065, respectively). The colonic concentration of iso-valeric acid was lowest in rabbits fed the SPM5 diet (*P* < 0.05 vs. SBM and SPM10).

**Table 6:** Concentrations [μmol/g digesta], profile [μmol/100 μmol total SCFA] and pool [μmol/kg BW] of SCFAs in the cecal and colonic digesta of rabbits (mean ± SEM^1^)

	Group^2^			*p value*
	SBM	SPM5	SPM10	
Cecum				
SCFA concentration				
acetic acid	32.5 ± 1.603	33.0 ± 2.028	29.8 ± 1.725	*0.283*
propionic acid	3.53 ± 0.305	4.60 ± 0.508	3.50 ± 0.185	*0.055*
iso-butyric acid	0.463 ± 0.045	0.443 ± 0.038	0.353 ± 0.018	*0.067*
butyric acid	2.95 ± 0.288^ab^	3.20 ± 0.358^a^	2.16 ± 0.198^b^	*0.033*
iso-valeric acid	0.713 ± 0.053^a^	0.688 ± 0.050^a^	0.525 ± 0.040^b^	*0.021*
valeric acid	0.585 ± 0.043^ab^	0.713 ± 0.128^a^	0.435 ± 0.038^b^	*0.040*
total putrefactive SCFAs	1.76 ± 0.120^a^	1.85 ± 0.180^a^	1.31 ± 0.068^b^	*0.018*
total SCFAs	40.8 ± 2.001	42.8 ± 2.775	36.8 ± 1.753	*0.110*
SCFA profile				
acetic acid	79.9 ± 1.006^ab^	77.6 ± 0.592^b^	80.9 ± 1.051^a^	*0.030*
propionic	8.64 ± 0.634	10.5 ± 0.519	9.64 ± 0.841	*0.094*
butyric acid	7.12 ± 0.415^ab^	7.58 ± 0.685^a^	5.86 ± 0.419^b^	*0.049*
SCFA pool	1573 ± 90.11	1900 ± 199.4	2055 ± 161.6	*0.067*
Colon				
SCFA concentration				
acetic acid	18.6 ± 1.385	16.8 ± 1.783	13.7 ± 1.538	*0.065*
propionic acid	2.38 ± 0.155	2.73 ± 0.220	2.14 ± 0.428	*0.222*
iso-butyric acid	0.343 ± 0.035	0.363 ± 0.023	0.405 ± 0.033	*0.226*
butyric acid	1.62 ± 0.125	1.74 ± 0.188	1.27 ± 0.195	*0.103*
iso-valeric acid	0.475 ± 0.053^a^	0.258 ± 0.043^b^	0.420 ± 0.030^a^	*0.003*
valeric acid	0.320 ± 0.025	0.333 ± 0.028	0.318 ± 0.018	*0.666*
total putrefactive SCFAs	1.14 ± 0.073	0.955 ± 0.053	1.14 ± 0.053	*0.055*
total SCFAs	23.7 ± 1.233	22.2 ± 1.920	18.3 ± 1.798	*0.053*
SCFA profile				
acetic acid	77.5 ± 2.303	74.3 ± 2.246	74.2 ± 2.667	*0.399*
propionic acid	10.4 ± 0.993	12.9 ± 1.228	11.7 ± 1.803	*0.278*
butyric acid	7.17 ± 1.006	8.28 ± 1.054	7.25 ± 1.095	*0.519*
SCFA pool	250 ± 11.72	280 ± 32.65	268 ± 44.63	*0.589*

^a, b^means with different superscripts in the same row differ significantly (*P* < 0.05)

^1^SEM - standard error of the mean

^2^Group: SBM - 10% SBM, SPM5–5% SBM and 5% SPM, SPM10–10% SPM (SBM - soybean meal, SPM - dried silkworm pupae meal)

An analysis of blood biochemical parameters indicated that both SPM treatments contributed to a significant increase in plasma albumin concentration (*P* < 0.05 vs. SBM, Table 7). Plasma urea concentration was significantly (*P* < 0.05) higher in group SPM10 than in treatments SBM and SPM5. The remaining biochemical parameters of blood plasma were not affected by the applied dietary treatments (*P* > 0.05).

**Table 7:** Blood plasma biochemical parameters of rabbits (mean ± SEM^1^)

	Group^2^			*p value*
	SBM	SPM5	SPM10	
AST [U/L]	19.7 ± 2.161	18.4 ± 0.832	22.1 ± 1.969	*0.208*
ALT [U/L]	23.9 ± 4.277	23.4 ± 1.102	24.7 ± 2.138	*0.783*
Creatinine [μmol/L]	40.5 ± 3.716	39.8 ± 3.696	50.4 ± 4.860	*0.121*
GGT [U/L]	11.8 ± 1.182	12.9 ± 1.016	12.8 ± 0.929	*0.529*
Albumin [μmol/L]	518 ± 24.20^b^	578 ± 16.59^a^	593 ± 11.96^a^	*0.016*
ALP [U/L]	204 ± 13.60	191 ± 14.14	166 ± 17.66	*0.132*
Urea [mmol/L]	5.92 ± 0.220^b^	6.00 ± 0.396^b^	7.34 ± 0.559^a^	*0.043*
Total protein [g/L]	58.7 ± 1.443	60.1 ± 0.821	59.7 ± 0.922	*0.459*

^a, b^means with different superscripts in the same row differ significantly (*P* < 0.05)

^1^SEM - standard error of the mean

^2^Group: SBM - 10% SBM, SPM5–5% SBM and 5% SPM, SPM10–10% SPM (SBM - soybean meal, SPM - dried silkworm pupae meal)

## Discussion

In general, the digestibility coefficients determined in this study are typical of growing meat-type rabbits [6, 26] and consistent with the results of our previous study investigating the effect of dietary supplementation with SPM on growth performance [19]. In the cited study, an increase in the SPM content of rabbit diets was accompanied by a decrease in the values of performance parameters such as final body weight, carcass weight and dressing percentage, which could be linked with nutrient (in particular protein) digestibility. The observed decrease in digestibility is difficult to interpret, but it could result from the presence of chitin in silkworm larvae. Herbivorous animal species such as rabbits and guinea pigs do not have functional acidic chitinase (Chia) genes, and therefore they are unable to digest chitin [27]. However, as reported by Suresh et al. [28], the amount of chitin in silkworm pupae is low, at around 3% on a DM basis. Therefore, the administered feed contained only 0.12% chitin. In a study of another herbivorous species, the chinchilla, diets supplemented with 4% dried mealworm *(Tenebrio molitor*) larvae meal had no influence on the digestibility of DM, organic matter, total protein, NDF and energy, but they improved the digestibility of ether extract, ADF and ADL [29]. Martins et al. [30] and Gasco et al. [26] found that black soldier fly (*Hermetia illucens* L.) larvae fat and mealworm fat had no adverse effects on nutrient digestibility in rabbits.

The calculated values of daily N balance remained within normal limits for rabbits aged 55–65 days [6, 7]. Lower N intake in the experimental groups corresponded to lower total feed intake during the production trial where rabbits were fed diets containing SPM [19], which is difficult to explain. This could result from the higher energy value or lower palatability of SPM diets. Experiments of the type have not been conducted on rabbits to date.

In groups SPM5 and SPM10, N excretion in feces and urine, digestion and retention decreased proportionally to N intake. Nitrogen retention relative to N intake and N digested corresponded to body weight gain and decreased with increasing inclusion levels of SPM in rabbit diets [19]. However, different results were reported by Kowalska et al. [20] who found that the 4% addition of dried silkworm pupae and mealworm larvae meals to rabbit diets increased their body weight gains, compared with the control group, which could be correlated with higher N retention.

In the present experiment, both SPM treatments caused an undesirable increase in gastric pH, which gives cause for concern and needs to be further investigated. It appears that this effect could be due to the higher fat content of SPM diets [31]. Stomach acidity plays an important role in breaking down food, particularly in denaturing proteins via pepsinogen and HCl. It also acts as a barrier to pathogen colonization [32]. Therefore, the increase in gastric pH noted in treatments SPM 5 and SPM10 could be one of the factors that negatively affected digestion processes and microbial activity in the large intestine.

Complete replacement of SBM with 10% SPM resulted in a tendency towards increased relative weight of the small intestinal contents. This could be partially due to the fact that diets containing SPM, in particular at the higher inclusion rate, were characterized by higher water holding capacity. The above effect was observed in the upper GIT as well as in the cecum and colon of SPM10 group rabbits, although feed intake was reduced in animals fed SPM diets [19]. Interestingly, the SPM treatments did not affect the apparent viscosity of the small intestinal digesta in rabbits. Increased viscosity of the intestinal contents weakens the effect of peristaltic mixing of digesta, lowers bile acid production and suppresses deconjugation processes, which delays the diffusion of nutrients through the intestinal wall and reduces the rate of digestion and absorption of organic nutrients [33]. The results of the present study indicate that SPM added at up to 10% to rabbit diets had a minor effect on the viscosity of the small intestinal digesta. However, such dietary treatments may contribute to more “watery” digesta (see a statistical tendency in jejunal DM percentage), therefore the above effect should be further investigated. The depressed growth rate of rabbits fed SPM diets, observed by Gugołek et al. [19], could be partially attributed to decreased jejunal DM concentration and enhanced dilution of *succus entericus* and bile. Gugołek et al. [7] demonstrated that enhanced hydration of the small intestinal digesta in rabbits fed diets containing dried distillers grains with solubles could decrease their growth rate by disturbing digestive processes. It should be stressed that in the present experiment, the DM content of the cecal and colonic digesta was not reduced by SPM, and none of the animals manifested symptoms of diarrhea. The values of pH and other GIT parameters, determined in rabbits in this study, are comparable with those reported by Chrastinová et al. [34].

In rabbits and most other herbivores, the large intestinal environment, which is the main site of bacterial fermentation, plays a very important role in the digestive system [25]. Therefore, in the present experiment, the parameters of microbial metabolism, including enzyme activity and SCFA concentrations, were determined in the cecal and colonic digesta. Fermentation processes in the large intestine affect the pH of digesta, which has a considerable influence on the growth and activity of gut microbiota. Reasonable acidification of digesta is considered beneficial to the overall health of the large intestine and the development of desirable gut microbes, whereas excessively alkaline digesta exerts the opposite effect by supporting the growth of undesirable microbial species. Quite surprisingly, in the current study significant differences in the pH of cecal digesta were found between two SPM treatments – the lowest value was noted in group SPM5 and the highest value was observed in group SPM10. In view of the research hypothesis, partial replacement of SBM with SPM in rabbit diets could be physiologically advisable whereas complete replacement raises certain doubts. In addition, taking into account the increased accumulation of digesta in the small intestine (statistical tendency), cecum (statistical significance) and colon (statistical tendency) of rabbits fed the SPM 10 (but not SPM 5) diet, it can be concluded that the higher dietary inclusion level of SPM should not be recommended. It cannot be excluded that rabbits fed SPM for a prolonged period of time and/or at high inclusion rates would experience gastrointestinal stasis. However, no symptoms of gastrointestinal stasis (very small or no pellets, small pellets in clear or yellowish mucus, loud, violent gurgles due to gas moving) [35] were observed in the current experiment – it was meticulously checked during the balance period when feces samples were collected. The increased bulk of digesta, caused by slow peristaltic movement, can be attributed to the presence of specific fiber compounds (probably hemicelluloses, cf. NDF content) and the high fat content of SPM.

The increase in cecal pH in group SPM10 rabbits was accompanied by reduced activity (in particular extracellular activity) of important bacterial enzymes. It is known that extracellular enzyme activity directly affects the rate of microbial digestion of nutrients and non-nutrients in the lower GIT [8]. In turn, the total activity of enzymes comprised of extracellular and intracellular activities reflects the types of bacteria and the counts of bacterial species in the digesta. Klewicka et al. [36] reported that enhanced activity of bacterial β-glucuronidase followed the undesired growth of intestinal *Escherichia coli* and *Clostridium* populations. The reduced enzymatic activity of intestinal microbiota and the subsequent decrease in SCFA concentrations, observed in the current study, could be partially attributed to the fact that insect meals may contain active bacteriostatic peptides [37]. The dietary inclusion of SPM at 5 and 10% did not enhance the extracellular or total activity of β-glucuronidase in the cecal and colonic digesta, despite the differences in cecal pH between the SPM treatments. It can be assumed that the higher inclusion rate of SPM (10%) suppressed fermentation processes in the cecum of rabbits, which was not observed when SPM was added to the diets at 5%. This hypothesis is confirmed by the fact that the activity of bacterial α-glucosidase, β-glucosidase, α-galactosidase, α-arabinofuranosidase, α-arabinopyranosidase and β-cellobiosidase was considerably lower in the cecal digesta of rabbits fed the SPM10 diet than in those fed the SPM5 diet. The activity of the above enzymes is associated with important physiological processes such as starch hydrolysis (α-glucosidase), hydrolysis of non-starch polysaccharides (NSPs) and degradation of cellulose (β-glucosidase), utilization of raffinose-family oligosaccharides (α-galactosidase), degradation of NSPs present in both cereals and protein sources used in this study, namely arabinans containing terminal arabinofuranoses as well as internal arabinopyranoses (both arabinosidases) [38, 39]. It should be noted that similar increasing and decreasing trends in bacterial enzyme activity in the colonic digesta, induced by dietary supplementation with 4% mealworm larvae meal, were observed in chinchillas [29].

Chitin, a polymer of N-acetyl-D-glucosamine (GlcNAc), is a major structural component in chitin-containing organisms such as crustaceans, insects and fungi. Tabata et al. [27] reported that acidic chitinase was highly expressed in the stomach tissues of mice, chickens and pigs, and that it was able to digest chitin in their GITs. Interestingly, both partial and complete replacement of SBM with SPM resulted in increased activity of bacterial NAGase in the cecal and colonic digesta. The above effect could be attributed to the adaptation mechanism of microbiota aiming at deriving additional energy through cecal and colonic fermentation of poorly digestible dietary compounds such as chitin. At least two enzymes, chitinase and NAGase, are needed for the digestion and assimilation of chitin, an indigestible polysaccharide contained in insect meals [21]. It should be noted that the release rate of bacterial NAGase in the cecum and colon of SPM5 and SBM rabbits was significantly enhanced along with increased extracellular and total activities of NAGase, pointing to adaptive efficient utilization of chitin in the lower GIT of rabbits fed SPM diets. According to some authors, efficient utilization of chitin in hens fed insect-based diets (with insect meal as a substitute for SBM) increased SCFA production [40]. Such an effect was not observed in the current study.

Complete replacement of SBM with SPM decreased SCFA concentrations in the cecum (propionate, butyrate, PSCFAs) and the colon (total SCFAs, mainly acetic acid) of rabbits, which was accompanied by changes in the intestines (reduced enzyme activity, in particular extracellular activity, mentioned above) and in the blood plasma (increased urea concentration). The changes in plasma urea levels could be attributed to the fact that gut microbiota effectively convert N to protein, and that considerable amounts of N are derived from blood urea [41]. It should be noted that in all groups, the proportions of acetic, propionic and butyric acids in the SCFA profile remained within the normal ranges for rabbits fed balanced diets, proposed by Bovera et al. [42].

Both dietary inclusion levels of SPM contributed to an increase in plasma albumin concentration. It should be stressed that total plasma protein concentration was not affected by the treatments. In this trial, all diets had comparable nutrient content, including dietary protein and major amino acids. The digestibility coefficient of crude protein did not differ among treatments, either. Moreover, the cecal concentrations of ammonia and PSCFAs did not increase in response to SPM, which suggests that the amount of undigested protein entering the cecum with passing digesta did not increase, either. These complex processes could be responsible for the absence of significant differences in total plasma protein levels between groups. Research has shown that total protein synthesis in the blood is related to the content of available protein in the diet [26]. In the present experiment, plasma albumin concentration increased in response to SPM, which should be considered beneficial. Albumins play important functions in the body, they e.g. participate in the transport of metals, fatty acids, cholesterol and bile, and regulate osmotic pressure. Albumins are also effective antioxidants in plasma whose components are highly exposed to reactive oxygen species [43]. It should be noted that also in other experiments, diets containing insect meals and other animal protein sources such as fishmeal had no influence on most serum biochemical parameters in rabbits [13, 26, 44].

## Conclusions

It can be concluded that partial replacement of SBM with 5% SPM did not disturb gastrointestinal physiology, whereas such an effect was observed when SPM was added to rabbit diets at 10%. Increasing inclusion levels of SPM caused a minor decrease in nutrient digestibility and a considerable decrease in N retention. The addition of 10% SPM to rabbit diets increased the bulk of the small intestinal, cecal and colonic digesta, decreased bacterial enzyme activity and SCFA concentrations in the cecum, and increased cecal pH. The results of this study indicate that rabbit diets can be supplemented with SPM at up to 5%.

## Methods

### Animals and housing

The experiment was performed on 30 male Termond White rabbits raised on a farm in southern Poland. The animals were randomly divided into three groups, analogous in terms of origin and body weight (*n* = 10). The average body weight of rabbits at the beginning of the digestibility trial, i.e. at 45 days of age, was 1017.62 ± SEM 41.88. The study was conducted in November, in a separate facility in a closed building where cages were placed. The animals were housed under standard conditions with a temperature of 16–18 °C, relative air humidity of 60–75%, forced room ventilation, and a controlled photoperiod (12 h light with intensity of 25 lx, and 12 h dark).

### Diets and experimental procedures

Control group rabbits were fed a diet containing 10% SBM. In the first experimental group (SPM5), rabbits received a diet containing 5% SBM and 5% dried SPM. The diet administered to the second experimental group (SPM10) was supplemented with 10% dried SPM. The ingredients of the diets are presented in Table 8, and the chemical composition of diets and experimental factors are presented in Table 9. All diets were iso-nitrogenous and their nutritional value corresponded to the requirements of growing meat-type rabbits [3].

**Table 8:** Composition of experimental diets [%]

Ingredients	Diet^1^		
	SBM	SPM5	SPM10
SBM^2^	10.0	5.0	0.0
SPM^3^	0.0	5.0	10.0
Dried alfalfa	20.0	20.0	20.0
Wheat bran	42.0	42.0	42.0
Rapeseed meal	6.0	6.0	6.0
DDGS^4^	6.0	6.0	6.0
Crude fiber concentrate (90%)	6.0	6.0	6.0
Dried beet pulp	5.0	5.0	5.0
Dried brewer’s yeast	1.0	1.0	1.0
Whey powder	1.0	1.0	1.0
Sodium chloride (NaCl)	0.2	0.2	0.2
Chalk	1.3	1.3	1.3
Phosphate	0.5	0.5	0.5
Mineral-vitamin premix^5^	1.0	1.0	1.0
Total	100.0	100.0	100.0

^1^Diet: SBM - 10% SBM, SPM5–5% SBM and 5% SPM, SPM10–10% SPM

^2^SBM - soybean meal

^3^SPM - dried silkworm pupae meal

^4^DDGS- dried distilled grains with solubles

^5^Composition of the mineral-vitamin premix (1 kg): vit. A – 3,500,000 IU, vit. D_3_–200000 IU, vit. E – 28,000 mg, vit. K_3_–200 mg, vit. B_1_–1500 mg, vit. B_2_–2800 mg, vit. B_6_–2800 mg, vit. B_12_–20000 mcg, folic acid – 200 mg, niacin – 10,000 mg, biotin – 200,000 mcg, calcium pantothenate – 7000 mg, choline – 30,000 mg, Fe – 17,000 mg, Zn – 2000 mg, Mn – 1000 mg, Cu (copper sulfate x 5H_2_O, 24,5%) – 800 mg, Co – 1000 mg, I – 100 mg, Ca – 150 g, P – 100 g

**Table 9:** Chemical composition of experimental diets [%]

	Diet^1^			SBM^2^	SPM^3^
	SBM	SPM5	SPM10		
Dry matter	88.70	88.84	89.14	88.68	93.19
Crude ash	7.41	7.31	7.20	6.02	3.93
Organic matter	81.29	81.53	81.94	82.66	89.26
Total protein	18.07	18.34	18.55	46.56	51.58
Ether extract	3.25	4.47	5.64	1.98	26.49
Neutral detergent fiber	28.99	29.98	31.09	10.63	31.35
Acid detergent fiber	16.26	16.45	16.71	6.25	9.34
Acid detergent lignin	5.12	5.23	5.29	1.15	2.95
Lysine	0.92	0.92	0.92	2.99	2.85
Methionine+cystine	0.87	0.90	0.92	1.32	1.78
Threonine	0.81	0.82	0.83	1.72	1.96
Tryptophan	0.21	0.22	0.22	0.68	0.74
Gross energy [MJ/kg]	16.31	16.74	17.24	17.36	24.69

^1^Diet: SBM - 10% SBM, SPM5–5% SBM and 5% SPM, SPM10–10% SPM

^2^SBM - soybean meal

^3^SPM - dried silkworm pupae meal

From 35 to 45 days of age, rabbits were kept in standard cages measuring 0.5 × 0.6 × 0.4 m (two animals per cage). From 45 to 65 days of age, during a digestibility-balance trial, the animals were housed in individual metabolism cages for quantitative urine and feces collection. The experiment was preceded by a 10-day adaptation period when the rabbits were allowed to adapt to the new diet and environmental conditions. Pelleted feed (150 g) was offered once daily, at 10 a.m. The animals had ad libitum access to water. Feces and non-ingested feed residues were collected on a daily basis, and were weighed to the nearest 1 g. The feces were frozen, and feces and feed samples were dried and ground. Urine was preserved with 20% sulfuric acid, and the total volume of collected urine was calculated at the completion of the experiment. Feed samples were also analyzed to determine their energy value and chemical composition.

In this study, the balance method was used to calculate the coefficients of nutrient and energy digestibility (DC) and nitrogen (N) retention. Nutrient digestibility was calculated using the following equation: DC = (a – b)/a × 100%, where: a - nutrient content of feed, b - nutrient content of feces.

After the completion of the digestibility-balance trial, rabbits were kept in the same housing facility in standard wire-mesh flat-deck cages until 90 days of age. They had ad libitum access to feed served once a day via automatic feeders and water from nipple drinkers.

Ninety-day-old rabbits were sacrificed after fasting for 24 h, in accordance with the recommendations for the euthanasia of experimental animals. Immediately before euthanasia, blood was collected from the ear vein to heparinized 2.5 mL test tubes. The procedure was supervised by a veterinarian. The gastrointestinal tracts of rabbits were analyzed at slaughter. After laparotomy, segments of the digestive tract (stomach, small intestine, cecum and colon) were dissected and weighed, including the contents. Fresh digesta samples were subjected to immediate analysis (pH, DM content, viscosity of the small intestinal digesta, concentrations of cecal ammonia and cecal/colonic short-chain fatty acids – SCFAs). The remaining digesta was stored in microfuge tubes at − 70 °C. The small intestine, cecum and colon were thoroughly flushed with ice-cold saline, blotted and dried on filter paper, and weighed.

### Analytical methods

Feed and feces samples were subjected to chemical analyses. The content of DM, crude ash, total protein, ether extract, acid detergent fiber (ADF) and acid detergent lignin (ADL) was determined by standard methods [45]. The content of neutral detergent fiber (NDF), ADF and ADL was estimated in the FOSS TECATOR Fibertec 2010 System; NDF content was determined according to the procedure proposed by Van Soest et al. [46]. Gross energy content was determined using a bomb calorimeter (IKA® C2000 basic, Germany).

As soon as possible after euthanasia (~ 15 min), the pH of the stomach, jejunum, cecum and colon was measured directly in the organs’ contents using a microelectrode and a pH/ION meter (model 301, Hanna Instruments, Vila do Conde, Portugal). The DM content of the samples (jejunum, cecum) was determined at 103 °C. Pooled samples of the small intestinal digesta were collected, vortexed and centrifuged at 7211 *g* for 10 min. The supernatant fraction (0.5 mL) was placed in the Brookfield LVDV-II+ cone-plate rotational viscometer (CP40; Brookfield Engineering Laboratories, Stoughton, MA, USA), and the viscosity of pooled samples was measured at a constant temperature of 37 °C and a shear rate of 60/s. Viscosity was recorded as apparent viscosity. Ammonia was extracted from fresh cecal digesta, trapped in a solution of boric acid in Conway dishes, and determined by direct titration with sulfuric acid [47].

The concentrations of SCFAs in samples of the cecal and colonic digesta were analyzed in a gas chromatograph (Shimadzu GC-2010, Kyoto, Japan). The samples (0.2 g) were mixed with 0.2 mL of formic acid, diluted with deionized water and centrifuged at 7211 *g* for 10 min. The supernatant was transferred to a vial and loaded onto a capillary column (SGE BP21, 30 m × 0.53 mm) using an on-column injector. Initial oven temperature was 85 °C, it was raised to 180 °C in steps of 8 °C/min, and maintained for 3 min. The temperature of the flame ionization detector and the injection port was 180 °C and 85 °C, respectively. The volume of the sample for gas chromatography was 1 μL. The concentrations of cecal/colonic putrefactive SCFAs (PSCFAs) were calculated as the sum of iso-butyrate, iso-valerate and valerate in the digesta. All SCFA analyses were performed in duplicate. Pure acetic, propionic, butyric, iso-butyric, iso-valeric and valeric acids were obtained from Sigma (Poznan, Poland), and they were combined to create a standard plot and calculate the amount of each acid. The additional set of pure acids was included in each GC run at five sampling intervals to maintain calibration.

In addition to SCFA analysis, cecal and colonic fermentation processes were also analyzed based on the activity of selected bacterial enzymes (*α*- and *β*-glucosidase, *α*- and *β*-galactosidase, *β*-glucuronidase, *α*-arabinopyranosidase, *α*-arabinofuranosidase, *β*-xylosidase, *β*-cellobiosidase and *N*-acetyl-*β*-D-glucosaminidase), which was measured as the rate of release of p-nitrophenol or o-nitrophenol from the respective nitrophenylglucosides (Sigma Co., Poznań, Poland), according to a previously described method [48]. The following substrates were used: p-nitrophenyl-α-D-glucopyranoside (for α-glucosidase), p-nitrophenyl-β-D-glucopyranoside (for β-glucosidase), p-nitrophenyl-α-D-galactopyranoside (for α-galactosidase), o-nitrophenyl- β-D-galactopyranoside (for β-galactosidase), p-nitrophenyl-β-D-glucuronide (for β-glucuronidase), p-nitrophenyl-α-L-arabinopyranoside (for α-arabinopyranosidase), p-nitrophenyl-α-L-arabinofuranoside (for α-arabinofuranosidase), p-nitrophenyl- β-D-xylopyranoside (for β-xylosidase), p-nitrophenyl- β-D-cellobioside (for β-cellobiosidase) and p-nitrophenyl-β-N-acetylglucosamide (for *N*-acetyl-*β*-D-glucosaminidase; NGAase). The activity of enzymes secreted by bacterial cells in the cecal and colonic environment was measured by preparing a reaction mixture containing 0.3 ml of the substrate solution (5 mM) and 0.2 mL of 1:10 (v/v) dilution of the cecal/colonic sample in 100 mM phosphate buffer (pH 7.0), which was centrifuged at 7211 *g* for 15 min. Incubation was carried out at 37 °C, and p-nitrophenol was quantified at 400 nm (o-nitrophenol was quantified at 420 nm) after the addition of 2.5 ml of 0.25 M-cold sodium carbonate. Enzyme activity was expressed in μmol of the product formed per hour per gram of fresh digesta. To determine the total activity of selected cecal and colonic bacterial enzymes, including extracellular activity (see the procedure above) and intracellular activity, a sample of cecal/colonic digesta diluted in phosphate buffer was mechanically disrupted by vortexing with glass beads (212–300 μm in diameter, four periods of 1 min each, with 1 min cooling intervals on ice) in the FastPrep®-24 homogenizer (MP Biomedicals, Santa Ana, Ca, US). The resulting mixture was centrifuged at 7211 *g* for 15 min at 4 °C. The supernatant was used for the enzyme assay described above. Intracellular enzyme activity was calculated by comparing total enzyme activity with the activity of bacterial enzymes released into the intestinal environment, and it was expressed in μmol of the product (PNP or ONP, p-nitrophenol or o-nitrophenol, respectively) formed per hour per gram of digesta. The respective calculation formulas were derived based on the model curves for PNP and ONP (PNP or ONP standard solution in 100 mM phosphate buffer, pH 7.0, 40 mg/L). Extracellular enzyme activity was also expressed as a percentage of total enzyme activity (the release rate of enzymes). All analyses were performed in duplicate.

Heparinized blood samples were centrifuged at 380×g in order to obtain blood plasma (MPW-352R; MPW MED. Instruments, Warsaw, Poland). The plasma was stored at − 70 °C until analyses. The following plasma biochemistry parameters were determined on an automatic analyzer (Pentra C200, Horiba Ltd., Kyoto, Japan): concentrations of total protein, urea, albumin, creatinine, and the activity of gamma-glutamyl transferase (GGT), asparagine aminotransferase (AST), alanine aminotransferase (ALT) and alkaline phosphatase (ALP).

### Statistical analyses

Data were expressed as means ± standard error of the mean (SEM). All results were analyzed with each rabbit as a replicate. The results were analyzed statistically by one-way ANOVA, and the significance of differences between groups was determined with Duncan’s multiple range test at a significance level of *p* ≤ 0.05. All calculations were performed in the Statistica 12.0 program [49].

## Data Availability

The datasets produced and analyzed during the current study are available from the corresponding author on reasonable request.

## Abbreviations

SPM: Silkworm pupae meal
SBM: Soybean meal
NAGase: N-acetyl-β-D-glucosaminidase
PSCFA: Putrefactive short-chain fatty acid
GIT: Gastrointestinal tract
DC: Digestibility coefficients
N: Nitrogen
DM: Dry matter
SCFA: Short-chain fatty acid
ADF: Acid detergent fiber
ADL: Acid detergent lignin
NDF: Neutral detergent fiber
GGT: Gamma-glutamyl transferase
AST: Asparagine aminotransferase
ALT: Alanine aminotransferase
ALP: Alkaline phosphatase
Chia: Acidic chitinase
NSPs: Non-starch polysaccharides
GlcNAc: N-acetyl-D-glucosamine
